# Longitudinal impact of extended-hours hemodialysis with a liberalized diet on nutritional status and survival outcomes: findings from the LIBERTY cohort

**DOI:** 10.1007/s10157-024-02602-7

**Published:** 2025-01-28

**Authors:** Takahiro Imaizumi, Masaki Okazaki, Manabu Hishida, Shimon Kurasawa, Nobuhiro Nishibori, Yoshihiro Nakamura, Shigefumi Ishikawa, Katsuhiko Suzuki, Yuki Takeda, Yuhei Otobe, Toru Kondo, Fumika Kaneda, Hiroshi Kaneda, Shoichi Maruyama

**Affiliations:** 1 Department of Nephrology, Nagoya University Graduate School of Medicine, 65 Tsurumai-Cho, Showa-Ku, Nagoya, Aichi 464-8550 Japan; 2 Department of Advanced Medicine, Nagoya University Hospital, Nagoya, Japan; 3 Department of Clinical Research Education, Nagoya University Graduate School of Medicine, Nagoya, Japan; 4 Kaikoukai Josai Hospital, Nagoya, Japan; 5 Department of Rehabilitation Science, Course of Physical Therapy, Osaka Metropolitan University, Habikino, Japan; 6 Department of Cardiology, Nagoya University Graduate School of Medicine, Nagoya, Japan; 7 Kamome Otsuko Clinic, Kitaibaraki, Japan; 8 Kamome Minatomirai Clinic, Yokohama, Japan

**Keywords:** Extended-hours hemodialysis, Liberalized diet, Longitudinal study, Protein-energy wasting

## Abstract

**Background:**

Protein-energy wasting (PEW), a unique weight loss linked to nutritional and metabolic abnormalities, is common in patients undergoing hemodialysis (HD) and associated with adverse outcomes. This study investigated whether extended-hours HD combined with a liberalized diet could overcome PEW and improve survival.

**Methods:**

The body mass index (BMI) and survival outcomes in patients undergoing extended-hours HD were evaluated for up to 8 years using data from the LIBeralized diet Extended-houRs hemodialysis Therapy (LIBERTY) cohort. Extended-hours HD was defined as weekly dialysis length ≥ 18 h.

**Results:**

The LIBERTY cohort included 402 patients who initiated extended-hours HD. An increase in the length and frequency of HD sessions was observed over time, with approximately 70% and 20% of patients undergoing extended-hours HD for > 21 h/week and > 3 sessions/week at 5 years, respectively. The BMI and percentage creatinine generation rate were maintained over time, with no substantial increase in the phosphorus and potassium levels. The estimated BMI initially increased, and thereafter plateaued over time in patients with a baseline BMI < 25 kg/m^2^, whereas it decreased gradually in patients with a baseline BMI ≥ 25 kg/m^2^ after several years from baseline. Ninety-one patients died, and 108 discontinued extended-hours HD during the median follow-up period of 6.2 years (interquartile range, 3.5–8.0), yielding a 5-year survival rate of 85%.

**Conclusions:**

Extended-hours HD with a liberalized diet may help achieve favorable survival outcomes and maintain nutritional status. Thus, it is a promising treatment option for managing PEW in patients undergoing HD.

**Supplementary Information:**

The online version contains supplementary material available at 10.1007/s10157-024-02602-7.

## Introduction

Protein-energy wasting (PEW), a unique weight loss associated with nutritional and metabolic abnormalities, is commonly observed in patients undergoing hemodialysis (HD) [1]. PEW is closely associated with mortality, hospitalization, and reduced quality of life (QOL) [2, 3]. Owing to its association with negative protein balance, PEW management traditionally centers on ensuring nutrient adequacy [4]. Uremic milieu, another potential underlying factor in PEW, has also been linked to altered protein metabolism [5–7]. Thus, improving the uremic milieu and providing adequate dietary intake simultaneously is necessary, given the complex etiology of PEW [6, 7].

Transition from conventional HD (typically 3 times weekly, 4 h/session) to longer treatment time and/or more frequent HD sessions allows for more enhanced removal of uremic solutes and better fluid management. This alternative HD regimen generally includes extended-hours HD/nocturnal HD (usually 3–4 times weekly, 6–8 h/session) and short daily HD (usually 5–7 times weekly, 1.5–3 h/session), performed either in-center or at home [8]. Numerous cohort studies have shown improvements in nutritional indicators [9, 10], phosphorus control [11], hypertension [11], left ventricular mass [12], erythropoiesis-stimulating agent (ESA) resistance [13], QOL [14], and overall survival outcomes in patients following alternative HD regimens [15–17]. However, the survival benefits reported by previous randomized controlled trials in patients receiving intensive HD have been conflicting. Furthermore, insufficient statistical power to detect survival benefits [12, 18–20] and bias toward selecting patients with preserved residual kidney function may have contributed to the failure to demonstrate the benefits of intensive HD regimens [21, 22].

Extended-hours HD, which can better approximate the physiology of native kidney function, could help address PEW in patients undergoing HD because it implements a combined strategy involving liberalized dietary intake [23, 24]. This strategy is also employed at our institution to prevent weight loss and cachexia among patients. The superiority of extended-hours HD over conventional HD has been reported in terms of survival, especially among older individuals [25]. Further, maintenance or increase in the body mass index (BMI) during the first year of extended-hours HD is associated with superior survival outcomes [26]. Thus, we hypothesized that a combined approach of extended-hours HD and liberalized diet may be suitable for overcoming PEW in patients undergoing maintenance HD.

Although extended-hours HD is associated with potential survival and nutritional benefits, patients undergoing extended-hours HD account for < 1% of those undergoing HD in Japan, where aging and undernutrition are prevalent [27]. This may be attributed to the lack of longitudinal real-world data on extended-hours HD. For example, the trajectory of the creatinine generation rate (CGR) may provide additional insights into the patient’s nutritional status as a surrogate for lean body mass and/or a warning sign of PEW [28, 29]. This study explored the longitudinal changes in HD prescriptions, estimated the trajectories of BMI and %CGR, and investigated the prognosis in patients undergoing extended-hours HD.

## Materials and methods

### Data source

An observational study platform, the LIBeralized diet Extended-houRs hemodialysis TherapY (LIBERTY) cohort, was established using data from the Medical Corporation Kamome Clinic, which comprises four dialysis facilities providing daytime and nocturnal extended-hours HD in the northeastern part of Japan. Three of these facilities are located in rural areas, and the fourth facility is located in the most populous city in Japan. The use of extended-hours HD and electronic medical records was commenced in 2006 and 2008, respectively, by the Medical Corporation Kamome Clinic.

Clinical data, including the demographic characteristics, HD conditions, prescriptions, and routine laboratory data, were extracted and anonymized. The LIBERTY cohort comprised a subset of prospective cohorts undergoing flash glucose monitoring, body composition assessments, and physical examinations (Supplementary Fig. 1). Blood samples were collected from the patients for research purposes. Only data from patients undergoing newly commenced extended-hours HD at the Medical Corporation Kamome Clinic between January 2008 and December 2017 were included.

Patients undergoing daytime extended-hours HD who regularly visited the clinics for ≥ 6 months with clinical data electronically available and those in whom the first available data were from ≤ 6 months after commencing extended-hours HD were included. The laboratory data; prescription data; and HD conditions, including frequency of HD sessions per week, duration of each session, pre- and post-HD body weight, blood flow rate, and ultrafiltration rate (UFR), were extracted. Data regarding demographic characteristics and outcomes (i.e., death and discontinuation of extended-hours HD) were collected and managed electronically. Patients are generally not permitted to undergo conventional HD at these HD centers. Patients planning to discontinue extended-hours HD are referred to other facilities.

### Dialysis treatment

The dialysis regimen evaluated in this study is a combination of liberalized diet and in-center daytime or nocturnal extended-hours HD. Extended-hours HD is defined as weekly dialysis session length ≥ 18 h. The Medical Corporation Kamome Clinic has two policies: expansion of permissible dietary choices and discretionary augmentation of the duration or frequency of HD, as needed. Improved management of solutes and fluids will facilitate the liberalization of daily diet. Liberalized diet was defined as the consumption of a diet similar to that of individuals without chronic kidney disease. For instance, all patients enrolled in this study were offered a normal intra-dialytic meal without strict potassium and phosphate restrictions during all HD treatment sessions. Patients who were undernourished were encouraged by physicians to increase their food intake for post-dialysis weight gain.

### Baseline and longitudinal data collection of dialysis conditions, prescriptions, and laboratory measurements

Data regarding the weekly duration and frequency of HD, blood flow rate, UFR, and BMI are summarized as mean values for the quarter. Most patients underwent three or four HD sessions per week; however, some underwent a fourth HD session every 2–4 weeks. Thus, the quarter mean HD hours in those who underwent > 3 HD sessions per week were calculated by summing the weekly dialysis hours for 3 months and dividing this sum by the number of weeks. Patients who underwent > 3 HD sessions per week were classified into the > 3 sessions per week category. The duration of conventional dialysis was defined as the interval between the commencement of conventional dialysis (peritoneal dialysis or HD) and the initiation of extended-hours HD. UFR (mL/h/kg) was calculated by dividing the amount of fluid (mL) removed per hour by the post-dialysis weight (kg) and averaged over a week. BMI was calculated based on the post-HD body weight.

The use of antihypertensive agents, ESAs, and phosphate binders was summarized every 3 months by determining whether these medicines were used within that period. The mean weekly doses of ESAs were calculated for each quarter. The number of classes of antihypertensive agents was defined as the frequency of using the following six categories of antihypertensive agents: alpha-blockers, beta-blockers, calcium-channel blockers, angiotensin-converting enzyme inhibitors or angiotensin receptor blockers, diuretics, and mineral corticoid receptor blockers. The ESA doses were converted from darbepoetin alfa to epoetin using a ratio of 1:200 [30]. ESA resistance index was calculated using the following equation: weekly ESA dose divided by [hemoglobin levels (g/dL) × body weight (kg)] [31, 32]. The quarter mean pre-dialysis levels of hemoglobin, albumin, potassium, and phosphorus were calculated. Data regarding the %CGR, single-pool Kt/V (spKt/V), and normalized protein catabolic rate (nPCR), calculated using previously reported formulae by Shinzato et al. [33, 34], were collected every 6 months. Data regarding HD conditions, medications, and laboratory parameters were collected and summarized every 3 months for up to 8 years after commencing extended-hours HD. The first available data were considered baseline values. The measurement assay for serum albumin was changed during the observational period despite the availability of the serum albumin values. This resulted in a slight decrease in the serum albumin levels. A longitudinal analysis of the serum albumin levels was not performed owing to the unavailability of an appropriate conversion formula.

### Outcomes

The longitudinal changes in BMI and %CGR were investigated as nutritional indicators, and BMI trajectories stratified according to baseline BMI were examined based on the obesity classification for Asians: < 18.5; 18.5 to < 22; 22 to < 25; and ≥ 25 kg/m^2^ [35]. In addition, the BMI trajectories stratified according to age, sex, diabetes mellitus (DM), and duration of conventional dialysis were also examined. BMI values were available in 393 of the 402 patients.

All-cause death for up to 8 years was evaluated using the Kaplan–Meier method. The patients were censored upon discontinuing extended-hours HD or on December 31, 2021 (administrative censoring). Extended-hours HD was discontinued for the following reasons: conversion to conventional dialysis; kidney transplantation; external factors, such as natural disasters, family issues, and relocation to other regions; and internal factors, such as nursing care requirement, difficulty in maintaining regular visits, and transfer to another facility owing to acute illness. Survival data were collected after discontinuation of extended-hours HD and transfer to other facilities. Patients who died < 3 months after transfer to other facilities were not classified as censored but as deceased.

### Statistical analysis

The baseline characteristics were summarized by stratifying according to the duration of conventional dialysis before commencing extended-hour HD (< 0.5 years, 0.5 to < 5 years, and ≥ 5 years). Continuous variables are presented as mean (standard deviation) or median (interquartile range [IQR]). Categorical variables are presented as numbers (%). Inter-group comparisons were performed using the chi-squared test for categorical variables and analysis of variance or Kruskal–Wallis test for continuous variables. Mixed-effects models with an unstructured variance–covariance matrix to account for within-subject correlation with a random intercept and slope, adjusted for baseline age, sex, DM, and duration of conventional dialysis, were used to compare the trajectories of BMI and %CGR among individuals categorized according to baseline BMI (< 18.5, 18.5 to < 22, 22 to < 25, and ≥ 25 kg/m^2^). Intergroup differences in the trajectories were evaluated using Wald’s test for the interaction term of BMI category according to visit. BMI and %CGR trajectories among the subgroups defined according to age (≥ 65 vs. < 65 years), sex, DM, and duration of conventional dialysis (< 0.5 years, 0.5 to < 5 years, and ≥ 5 years) were also compared, after adjusting for age, sex, DM, and duration of conventional dialysis, excluding variables specific to each stratum. Similarly, the trajectories in the deceased and living patients were also compared. Survival outcomes stratified according to duration of conventional dialysis were analyzed using the age- and sex-adjusted Kaplan–Meier method. Statistical significance was set at *P* < 0.05. All statistical analyses were performed using Stata MP 18.0 (StataCorp., TX, USA).

## Results

### Patient selection and baseline characteristics

After exclusion, the final analysis was conducted in 402 patients who initiated daytime extended-hours HD (Supplementary Fig. 2). Patients who had been on conventional dialysis for < 0.5 years were older, more likely to be men, and had a history of DM and cardiovascular disease. Moreover, the %CGR, nPCR, spKt/V, albumin, hemoglobin, potassium, and phosphorus levels were lower in these patients. Furthermore, these patients also received more antihypertensive medications and ESA at baseline (Table 1). Conversely, patients who had been on conventional dialysis for ≥ 5 years were more likely to undergo HD at an urban center and receive phosphate binders.

**Table 1:** Baseline characteristics

	Total (*N* = 402)	Duration of conventional dialysis (years)			
		< 0.5 (*n* = 238)	0.5 to < 5 (*n* = 83)	≥ 5 (*n* = 81)	*P* value
Baseline age, years	60 (14)	64 (14)	55 (15)	55 (12)	< 0.001
Male sex	277 (69%)	174 (73%)	57 (69%)	46 (57%)	0.023
Diabetes mellitus	185 (46%)	144 (61%)	29 (35%)	12 (15%)	< 0.001
History of cardiovascular disease	107 (27%)	77 (32%)	18 (22%)	12 (15%)	0.004
Urban facility	127 (32%)	26 (11%)	44 (53%)	57 (70%)	< 0.001
Arteriovenous fistula	386 (96%)	226 (95%)	81 (98%)	79 (98%)	0.42
Duration of conventional dialysis, years	0.1 (0–2.6)	0 (0–0.1)	1.8 (1.1–2.5)	11.3 (8.0–17.0)	< 0.001
Body mass index, kg/m^2^	22.9 (4.2)	23.2 (4.0)	22.8 (5.2)	21.8 (3.8)	0.047
< 18.5	48 (12%)	21 (9%)	13 (16%)	14 (19%)	0.048
18.5 to < 22	140 (36%)	81 (34%)	32 (39%)	27 (37%)	
22 to < 25	106 (27%)	73 (31%)	14 (17%)	19 (26%)	
≥ 25	99 (25%)	63 (26%)	23 (28%)	13 (18%)	
Baseline dialysis conditions					
Hours per week, hours	19 (18–23)	18 (18–21)	21 (19–24)	22 (18–24)	< 0.001
Ultrafiltration rate, mL/h/kg	6.8 (4.9–9.0)	5.5 (3.8–7.9)	7.7 (6.1–9.2)	8.1 (6.6–10.1)	< 0.001
Blood flow rate, mL/min	125 (110–145)	119 (100–130)	140 (123–150)	140 (130–150)	< 0.001
Post dialysis weight, kg	60.9 (14.0)	61.9 (13.5)	61.7 (16.2)	57.3 (12.5)	0.034
Sessions per week, times					0.071
3 times	367 (91%)	214 (90%)	81 (98%)	72 (89%)	
> 3 times	35 (9%)	24 (10%)	2 (2%)	9 (11%)	
Baseline laboratory data					
%Creatinine generation rate, %	95 (70–113)	81 (61–97)	106 (89–124)	118 (101–141)	< 0.001
Normalized protein catabolic rate, g/kg/day	0.87 (0.21)	0.79 (0.18)	0.94 (0.18)	0.99 (0.21)	< 0.001
Single-pool Kt/V	1.46 (1.23–1.77)	1.33 (1.12–1.56)	1.67 (1.37–1.88)	1.72 (1.52–1.95)	< 0.001
Potassium, mEq/L	4.7 (0.7)	4.5 (0.7)	5.0 (0.7)	5.1 (0.7)	< 0.001
Hemoglobin, g/dL	10.0 (1.3)	9.6 (1.3)	10.5 (1.2)	10.4 (1.1)	< 0.001
Albumin, g/dL	3.5 (0.4)	3.4 (0.4)	3.8 (0.3)	3.7 (0.3)	< 0.001
Phosphorus, mg/dL	5.3 (1.4)	5.1 (1.3)	5.5 (1.5)	5.6 (1.4)	0.011
Baseline medication					
Antihypertensive agents	304 (76%)	194 (82%)	57 (69%)	53 (65%)	0.004
Number of antihypertensive agents					< 0.001
0	98 (24%)	44 (19%)	26 (31%)	28 (35%)	
1	76 (19%)	35 (15%)	20 (24%)	21 (26%)	
2	94 (23%)	59 (25%)	18 (22%)	17 (21%)	
≥ 3	134 (33%)	100 (42%)	19 (23%)	15 (19%)	
RAS inhibitors	191 (48%)	128 (54%)	27 (33%)	36 (44%)	0.003
ESAs	313 (78%)	192 (80.7)	60 (72.3)	61 (75%)	0.24
ESA weekly dose	3000 (1300–5100)	3700 (1600–6100)	2250 (200–4100)	2300 (750–4100)	< 0.001
ESA resistance index, units per week/kg/(g/dL)	4.9 (2.0–9.9)	6.4 (2.6–11.4)	3.6 (0.2–7.1)	4.0 (1.0–7.5)	< 0.001
Phosphate binders	198 (49%)	69 (29%)	61 (74%)	68 (84%)	< 0.001

Data are expressed as mean (SD) or median (IQR) for continuous variables and n (%) for categorical variables. Statistical significance is set at *P* < 0.05. The dose of ESAs represents an EPO-equivalent dose

*RAS* renin-angiotensin system, *ESA* erythropoiesis-stimulating agent

### Longitudinal dialysis-related parameters, laboratory parameters, and medication use

**Fig. 1:**
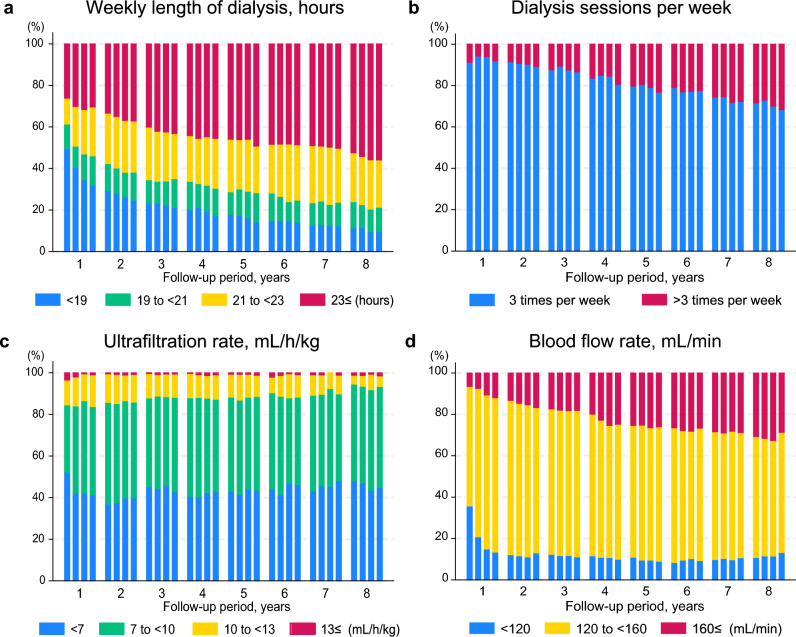
Longitudinal changes in dialysis conditions. **a** Weekly duration of dialysis (hours). **b** Dialysis sessions per week. More than three times including four times weekly, biweekly, and monthly. **c** Ultrafiltration rate (mL/h/kg). **d** Blood flow rate (mL/min). Each bar represents a quarterly category for each year up to 8 years. The quarterly categories for continuous variables are derived from the quarter means of each variable. Although the duration of weekly dialysis sessions, the number of sessions per week, and blood flow rate increase gradually, ultrafiltration rate remains largely unchanged

The duration and frequency of HD sessions per week were increased over time (Fig. 1). The UFR was < 7 mL/h/kg in approximately 40% of patients; among them, > 80% had UFR < 10 mL/h/kg. Patients with UFR ≥ 13 mL/h/kg were rare. The duration and frequency of HD gradually increased over time, and the UFR remained largely unchanged.

**Fig. 2:**
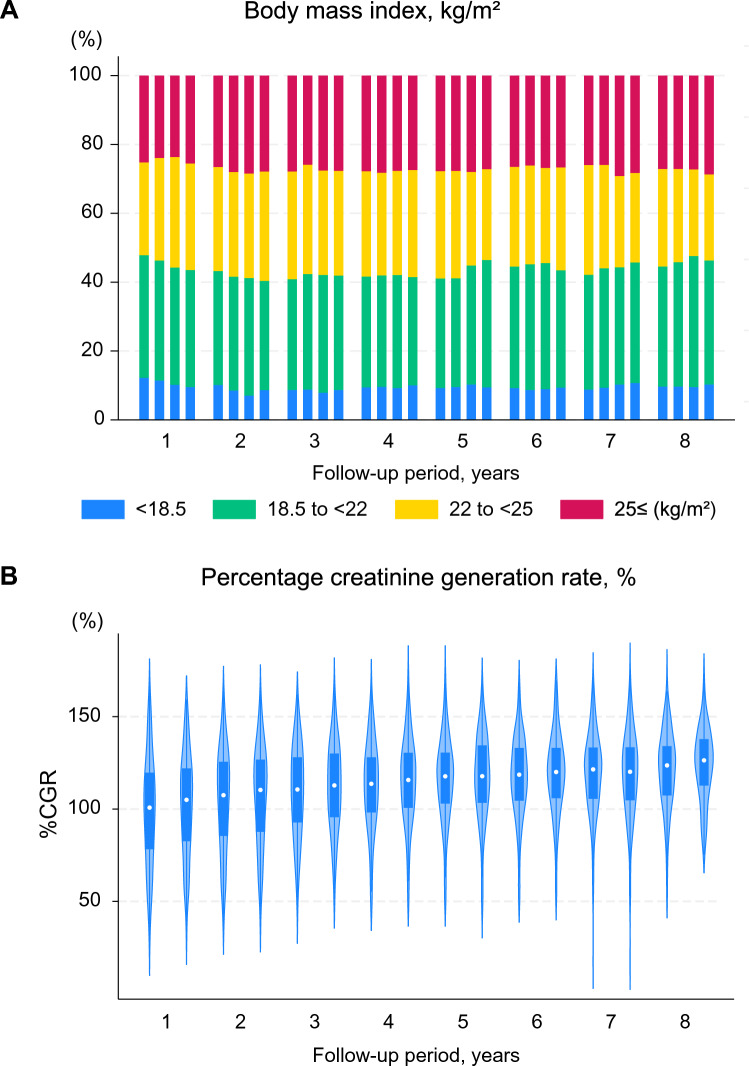
Longitudinal changes in nutritional parameters: body mass index and percentage creatinine generation rate. **a** Body mass index. **b** Percentage CGR. Each bar or violin plot represents a value or category summarized quarterly for each year up to 8 years. The quarterly categories of body mass index are derived from the quarter means of each variable. The mean values of %CGR are derived from the closest of the two annual measurements. Both parameters are well maintained over time. CGR, creatinine generation rate; BMI, body mass index

**Fig. 3:**
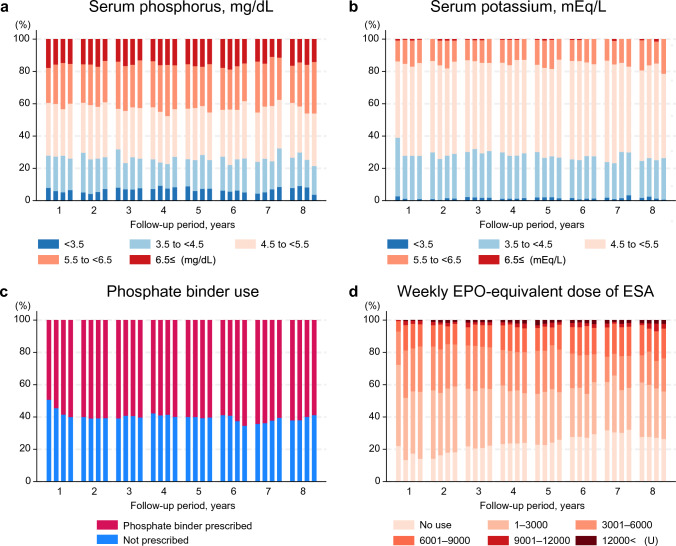
Longitudinal changes in laboratory parameters and medications in incident cases of extended-hours hemodialysis. **a** Serum phosphorus. **b** Serum potassium. **c** Phosphate binders. **d** Weekly dose of ESAs. The dose of ESAs represents an EPO-equivalent dose. Each bar represents a quarterly category for each year through 8 years. The quarterly categories for continuous variables are derived from the quarter means of each variable. Quarterly medications are defined as medications administered at least once in each quarter. The levels of phosphorus and potassium are stable and the proportion of ESA-free patients increased over time. The use of phosphate binder remains largely unchanged except initial increase in the first year. ESA, erythropoiesis-stimulating agent; EPO, erythropoietin

The BMI was well-maintained regardless of the duration of conventional dialysis (Fig. 2a; Supplementary Fig. 3). The proportion of patients with BMI < 18.5 kg/m^2^ gradually declined over time among those who had been undergoing conventional dialysis for ≥ 5 years (Supplementary Fig. 3d). The nPCR was also maintained over time** (**Fig. 2b). The serum phosphorus levels and proportion of patients receiving phosphate binders remained stable over time, except for an initial increase in the use of phosphate binders during the first year (Fig. 3). The serum potassium levels also remained stable over time, with appropriate levels being observed in > 50% of patients. The proportion of ESA-free patients increased from 15 to 30% over time. The number of classes of antihypertensive agents used also decreased over time (Supplementary Fig. 4). The baseline median [IQR] ERI value was 4.9 [2.0–9.9] and the ERI remained largely unchanged over time (Supplementary Fig. 5).

### Estimated trajectories of BMI and %CGR

**Fig. 4:**
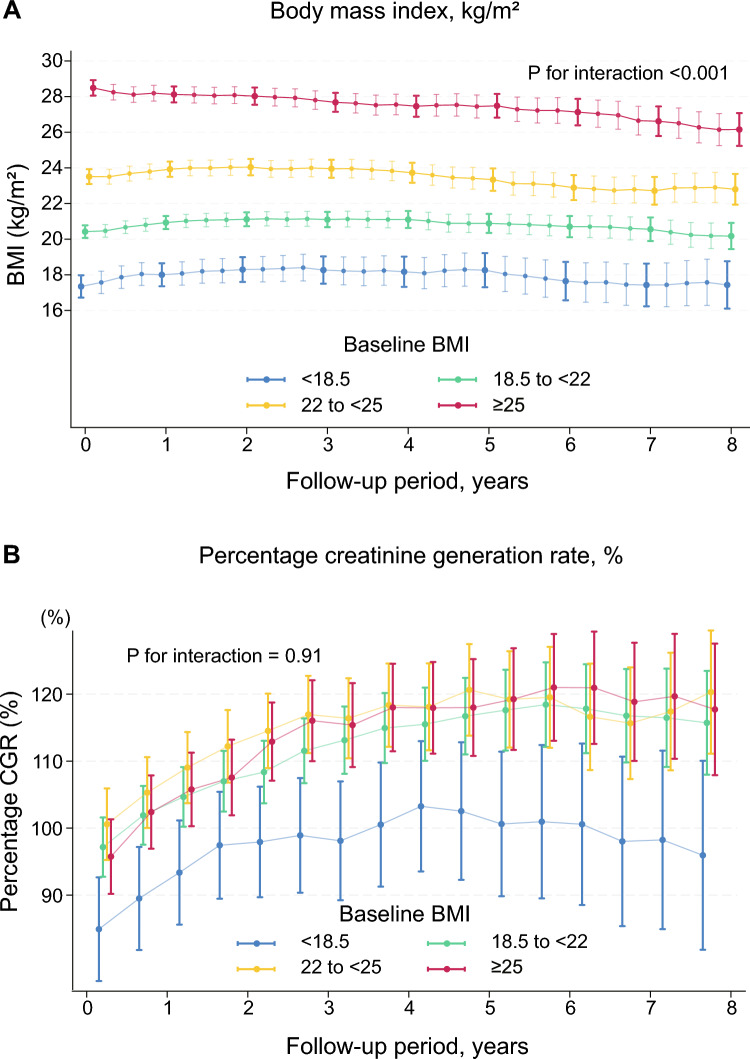
Estimated trajectories of body mass index and percentage creatinine generation rate stratified according to the initial values of body mass index. **a** BMI. **b** %CGR. The trajectories are estimated using a mixed-effects model with an unstructured variance–covariance matrix, adjusted for baseline age, sex, DM, and the duration of conventional dialysis, stratified according to the category of the baseline values of BMI: < 18.5; 18.5 to < 22; 22 to < 25; and ≥ 25 kg/m^2^. BMI is well preserved in patients with normal to subnormal baseline levels. The %CGR values increase over time regardless of the BMI category. The values increase even in patients with the lowest category of BMI. BMI, body mass index; CGR, creatinine generation rate; DM, diabetes mellitus

The BMI of patients with the highest baseline BMI gradually decreased over time, whereas that of patients in the other three categories increased slightly during the first few years, plateaued, and was then maintained over time (Fig. 4a). No significant differences were observed between the trajectories of men and women (P for interaction = 0.063; Supplementary Fig. 6). The estimated BMI increased during the first 2–3 years after enrolment and then declined rapidly in older patients. In contrast, the estimated BMI was largely maintained in younger patients (P for interaction < 0.001). Patients with DM initially presented with a higher BMI; however, the BMI declined gradually approximately 5 years after enrolment (P for interaction = 0.011). No significant differences were observed in the BMI trajectory in terms of the duration of conventional dialysis (P for interaction = 0.81). An initial (first 2 years of enrolment) increase in BMI was observed even in patients who had been undergoing conventional dialysis for ≥ 5 years. The estimated BMI of deceased and living patients was also compared (Supplementary Fig. 7). The BMI trajectory of deceased patients declined 2–3 years after commencing extended-hours HD; conversely, the trajectory among living patients remained largely unchanged over time (P for interaction < 0.001). The %CGR initially increased and then plateaued regardless of the initial BMI category (Fig. 4), sex, and age groups (Supplementary Fig. 8). Notably, the values increased even in patients with the lowest BMI category (Fig. 4b) and older individuals aged ≥ 65 years (Supplementary Fig. 8b).

### Overall survival and discontinuation of extended-hours HD

**Fig. 5:**
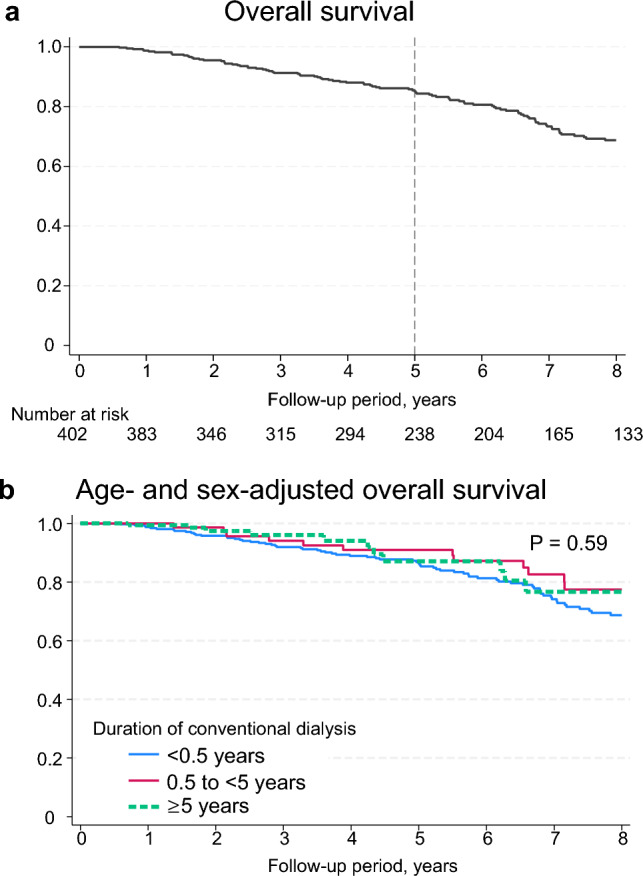
Overall survival. **a** Overall survival in all patients. Five-year survival is 0.85. **b** Age- and sex-adjusted overall survival, stratified according to the duration of conventional dialysis

Ninety-one patients died, and 108 discontinued extended-hours HD and were transferred to other facilities over a median duration of 6.2 [IQR, 3.5–8.0] years. The overall mortality rate was 4.1 per 100 person-years. The overall survival 5 years from the baseline was 85% (Fig. 5a). The overall survival rate did not differ based on the duration of conventional dialysis prior to inclusion in the cohort after adjusting for age and sex (Fig. 5b). Approximately one-third of the 108 patients who discontinued extended-hours HD discontinued treatment owing to personal preference (36%) and internal factors (32%) (Supplementary Table 2).

## Discussion

**Fig. 6:**
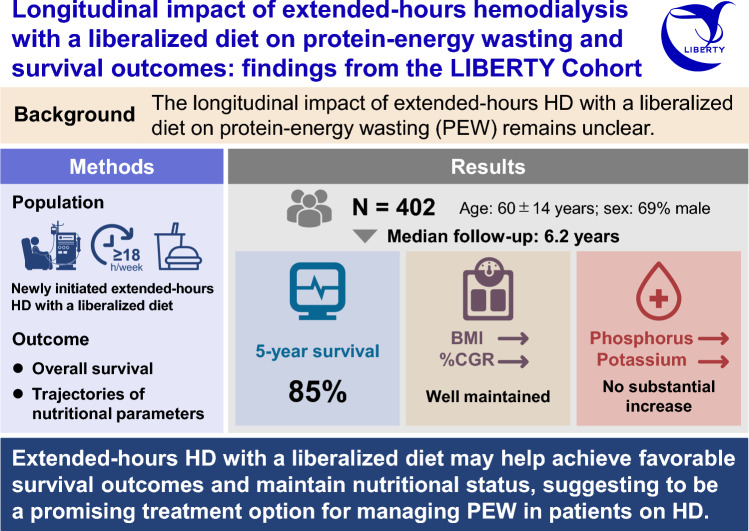
Graphical overview of the present study

Patients who commenced extended-hours HD with a liberalized diet had favorable survival outcomes, well-maintained BMI and other nutritional indices, and received decreased doses of ESAs and antihypertensive agents over time (Fig. 6). Consistent findings were observed even in patients who had been on conventional dialysis for > 5 years. In addition, the BMI was maintained over time in patients who initially presented with normal to subnormal BMI, and a similar trend was observed in those who had been undergoing conventional dialysis for a long duration, suggesting that extended-hours HD with a liberalized diet may prevent cachexia progression. The 5-year survival rate was impressive in patients who commenced extended-hours HD after undergoing conventional dialysis. The LIBERTY cohort is the first large-scale longitudinal observational study of extended-hours HD in Japan.

The international prospective in-center HD cohort of The Dialysis Outcomes and Practice Patterns Study (DOPPS) reported that the 5-year survival rate in patients undergoing HD in Japan was > 60% across different types of kidney disease [36, 37]. As indicated by the aging of the Japanese DOPPS participants in recent years, Japanese patients undergoing dialysis may be at increased risk of mortality, particularly due to infection-related deaths [38]. However, the 5-year survival rate was > 80% in patients undergoing extended-hours HD in the LIBERTY cohort. The mortality rate of patients with extended HD hours was 6–8 per 100 patient-years, which is superior to that in patients undergoing conventional HD (13–15 per 100 patient-years) [17, 39]. However, the follow-up duration of these studies without withdrawal from extended-hours dialysis regimen was substantially shorter than that in the present study (mean 2.5 years and median 7.6 months, respectively). Moreover, the mortality rates were also higher than the rate of 4.1/100 person-years observed in the present study. A decrease in hypertensive medication and ESA dosage was observed in the present study, consistent with previous findings [16, 40]. However, except for an initial increase in phosphate binder use within a year, the phosphorus levels and phosphate binder use remained almost constant over time in the present study, unlike those in previous studies [11]. This may be attributed to better maintenance of the nutritional status owing to the liberalized diet being prioritized over phosphorus removal during the initial phase.

The maintenance of %CGR and nPCR over time, with BMI trajectories being well-maintained in patients with normal to subnormal baseline BMI, indicated maintenance of nutritional status. Considering the pathophysiology that older patients undergoing dialysis are easily prone to PEW and/or cachexia, it is noteworthy that their post-dialysis body weight was maintained for several years (Supplementary Fig. 6). Similar to nPCR, which is a surrogate for dietary protein intake in stable patients, %CGR, which is a surrogate for muscle mass [28, 41], was well maintained over time in our LIBERTY cohort (Fig. 4b). Although the estimation of CGR using %CGR may be underestimated in patients with some residual kidney function (e.g., patients with their dialysis history within 2 years) [34], our findings suggest that extended-hours HD with liberalized diets may have long-term potential nutritional benefits in individuals on kidney replacement therapy. The simultaneous achievement of adequate fluid removal at a moderate UFR without hemodynamic instability and improvement of the uremic milieu, in addition to adequate dietary intake, may contribute to improvements in survival and nutritional parameters [42]. Although an increase in dialysis time and frequency was observed in the present study, UFR remained largely unchanged, indicating an increase in the dialysis volume and dietary intake. The BMI in patients who had a high baseline BMI, i.e., ≥ 25 kg/m^2^, gradually decreased toward the normal range, potentially due to increased physical activity resulting from an improved uremic milieu, which may lead to weight optimization.

Middle-molecular-weight toxins can exacerbate cardiovascular complications owing to vascular damage [43] and induce frailty via inflammation [44]. Removal of these toxins is limited by the dilution technique without convection, even with high-flux dialyzers [45, 46]. However, enhanced removal of uremic toxins and better fluid management in extended-hours HD may break the vicious cycle associated with PEW and inflammation in patients on HD [47]. Nationwide studies including DOPPS and the Japanese Society for Dialysis Therapy annual survey reported that a longer HD treatment time adjusted for Kt/V_urea_ was associated with better survival [48–50]. Extending the duration of HD facilitated adequate fluid removal, allowing both increased dietary intake and adequate removal of uremic retention solutes.

The present study has certain limitations. First, we did not compare the clinical parameters with those of patients undergoing conventional HD as the medical group does not offer conventional HD. In the future, we will include a pooled cohort with conventional HD for a comparative study. Second, since this was an observational study, selection bias may have been introduced owing to the preferences of patients and treatment indications. However, the three northern facilities were located in areas with limited healthcare resources. Moreover, some patients had difficulty accessing facilities providing conventional HD. Thus, most patients continued to undergo the same treatment. Third, nutritional status may appear to have been preserved during long-term observation owing to the effect of survival bias caused by the death of patients with poor nutritional status or severe morbid obesity. However, although the survival bias may have had an impact, nutritional indices were maintained 1–2 years after commencing observation, with few dropouts. Fourth, since regular intra-dialytic food consumption may affect pre- and/or post-dialysis urea levels [51], actual dietary protein intake may be underestimated by using Shinzato's nPCR equation in persons undergoing extended-hours HD [52, 53]. Validating whether Shinzato's nPCR indicates daily dietary protein intake in patients who usually eat full meals during extended-hours HD treatment is an important issue for future clinical research. Fifth, the clinical data prior to the transition to the extended-hours HD are not available. The trajectories of %CGR initially increased in all BMI categories, even in the subgroup with a dialysis vintage of ≥ 5 years (Fig. 4**; **Supplementary Fig. 8d). Despite the lack of data before the transition to extended-hours HD, our findings of long-term trajectories of nutritional parameters which contribute to better survival suggest that the transition from conventional HD to extended-hours HD might be beneficial for survival of patients with end-stage kidney disease [11, 16, 25, 26, 39]. Finally, objective information on fluid management, such as human atrial natriuretic peptide levels and cardiothoracic ratio on chest radiographs, could not be obtained. Further investigations on fluid management and cardiovascular events are warranted.

## Conclusion

Extended-hours HD with a liberalized diet may be more suitable for improving dialysis-related parameters and overcoming PEW. In particular, this treatment strategy could be an alternative to conventional dialysis in patients with long-term wasting conditions.

## Supplementary Information

Below is the link to the electronic supplementary material.

Supplementary file1 (DOCX 1683 KB)
