# Supplementary material for: Longitudinal impact of extended-hours hemodialysis with a liberalized diet on nutritional status and survival outcomes: findings from the LIBERTY cohort
Source: Clin Exp Nephrol. 2025 Jan 28;29(6):818–30. doi: 10.1007/s10157-024-02602-7 (PMC12125144; doi:10.1007/s10157-024-02602-7)
Supplement: Supplementary file 1 — Supplementary file1 (DOCX 1683 KB) [file 10157_2024_2602_MOESM1_ESM.docx]

**Longitudinal Impact of Extended-Hours Hemodialysis with a Liberalized Diet on Nutritional Status and Survival Outcomes: Findings from the LIBERTY Cohort**

**Supplementary Material**

**Authors:**

Takahiro Imaizumi, MD, PhD, Masaki Okazaki, MD, PhD, Manabu Hishida, MD, PhD, Shimon Kurasawa, MD, PhD, Nobuhiro Nishibori, MD, Yoshihiro Nakamura, MD, Shigefumi Ishikawa, MD, Katsuhiko Suzuki, MD, Yuki Takeda, MD, Yuhei Otobe, RPT, PhD, Toru Kondo, MD, PhD, Fumika Kaneda, MD, Hiroshi Kaneda, MD, Shoichi Maruyama, MD, PhD*

***Corresponding author:**

Email: [marus@med.nagoya-u.ac.jp](mailto:marus@med.nagoya-u.ac.jp)

Supplementary material

[**Supplementary Table 1.** Characteristics of patients across categories of body mass index (N = 393) 2](#_Toc169960161)

[**Supplementary Table 2.** Reasons for cessation of extended-hours hemodialysis 4](#_Toc169960162)

[**Supplementary Fig 1.** Schedule of the LIBERTY cohort 5](#_Toc169960163)

[**Supplementary Fig 2.** Flow diagram 6](#_Toc169960164)

[**Supplementary Fig 3.** Longitudinal changes in body mass index stratified by duration of conventional dialysis 7](#_Toc169960165)

[**Supplementary Fig 4.** Longitudinal changes in the number of antihypertensive agents 8](#_Toc169960166)

[**Supplementary Fig 5.** Longitudinal change in erythropoiesis-stimulating agent resistance index 9](#_Toc169960167)

[**Supplementary Fig 6.** The estimated trajectories of body mass index across different strata 10](#_Toc169960168)

[**Supplementary Fig 7.** The estimated trajectories of body mass index between deceased and living patients 11](#_Toc169960169)

[**Supplementary Fig 8.** The estimated trajectories of percentage creatinine generation rate across different strata 12](#_Toc169960170)

Supplementary Table 1. Characteristics of patients across categories of body mass index (N = 393)

|  | BMI<18.5  (n = 48) | 18.5 to <22  (n = 140) | 22 to <25  (n = 106) | ≥25  (n = 99) | P value |
| --- | --- | --- | --- | --- | --- |
| Baseline age, year | 61 (17) | 60 (15) | 65 (13) | 55 (12) | <0.001 |
| Male sex | 18 (38%) | 101 (72%) | 83 (78%) | 71 (71%) | <0.001 |
| Diabetes mellitus | 12 (25%) | 55 (39%) | 51 (48%) | 64 (65%) | <0.001 |
| History of cardiovascular disease | 11 (23%) | 27 (19%) | 36 (34%) | 31 (31%) | 0.042 |
| Urban facility | 21 (44%) | 51 (36%) | 23 (22%) | 24 (24%) | 0.007 |
| Arteriovenous fistula | 46 (96%) | 138 (99%) | 100 (94%) | 93 (94%) | 0.24 |
| Duration of conventional dialysis, years | 1.1 (0.1–7.1) | 0.2 (0.0–2.2) | 0.1 (0.0–2.1) | 0.0 (0.0–1.8) | <0.001 |
| Body mass index, kg/m^2^ | 17.3 (1.1) | 20.4 (1.0) | 23.4 (0.8) | 28.6 (3.4) | <0.001 |
| Baseline dialysis conditions |  |  |  |  |  |
| Hours per week, hours | 20 (18–24) | 20 (18–24) | 18 (18–23) | 18 (18–21) | 0.028 |
| Ultrafiltration rate, mL/h/kg | 8.9 (6.4–11.3) | 7.5 (5.3–9.3) | 6.2 (4.1–7.8) | 5.4 (3.9–7.5) | <0.001 |
| Blood flow rate, ml/min | 122 (100–140) | 121 (110–140) | 122 (114–150) | 128 (106–150) | 0.87 |
| Post dialysis weight, kg | 43.5 (5.3) | 54.4 (6.9) | 62.2 (6.8) | 78.2 (11.7) | <0.001 |
| Sessions per week, times |  |  |  |  | 0.79 |
| 3 times | 42 (88%) | 128 (91%) | 98 (92%) | 90 (91%) |  |
| >3 times | 6 (12%) | 12 (9%) | 8 (8%) | 9 (9%) |  |
| Baseline laboratory data |  |  |  |  |  |
| %Creatinine generation rate, % | 90 (77–104) | 95 (70–112) | 97 (82–115) | 83 (58–108) | 0.17 |
| Normalized protein catabolic rate, g/kg/day | 0.97 (0.27) | 0.90 (0.19) | 0.83 (0.18) | 0.79 (0.20) | <0.001 |
| Single-pool Kt/V | 1.70 (1.54–2.06) | 1.58 (1.30–1.87) | 1.40 (1.23–1.67) | 1.22 (1.07–1.41) | <0.001 |
| Potassium, mEq/L | 4.8 (0.8) | 4.7 (0.7) | 4.7 (0.7) | 4.7 (0.7) | 0.97 |
| Hemoglobin, g/dL | 10.1 (1.3) | 9.9 (1.4) | 9.9 (1.2) | 10.0 (1.2) | 0.58 |
| Albumin, g/dL | 3.6 (0.5) | 3.5 (0.4) | 3.5 (0.4) | 3.5 (0.5) | 0.82 |
| Phosphorus, mg/dL | 5.2 (1.4) | 5.1 (1.1) | 5.3 (1.4) | 5.7 (1.6) | 0.013 |
| Baseline medication |  |  |  |  |  |
| Antihypertensive agents | 29 (60%) | 102 (73%) | 88 (83%) | 79 (80%) | 0.013 |
| Number of antihypertensive agents |  |  |  |  | 0.028 |
| 0 | 19 (40%) | 38 (27%) | 18 (17%) | 20 (20%) |  |
| 1 | 11 (23%) | 22 (16%) | 22 (21%) | 20 (20%) |  |
| 2 | 7 (15%) | 40 (29%) | 24 (23%) | 19 (19%) |  |
| ≥3 | 11 (23%) | 40 (29%) | 42 (40%) | 40 (40%) |  |
| RAS inhibitors | 17 (35%) | 62 (44%) | 57 (54%) | 49 (50%) | 0.16 |
| ESA | 30 (63%) | 111 (79%) | 87 (82%) | 78 (79%) | 0.049 |
| ESA weekly dose | 500 (0–3200) | 1600 (400–3200) | 1700 (500–3600) | 1300 (300–3100) | 0.10 |
| ESA resistance index, units per week/kg/(g/dL) | 6.9 (3.4–14.4) | 5.9 (2.5–10.6) | 4.8 (1.8–9.2) | 3.5 (0.5–7.5) | 0.003 |
| Phosphate binders | 25 (52%) | 70 (50%) | 48 (45%) | 47 (48%) | 0.84 |

Data are expressed as mean (SD) or median (IQR) for continuous variables and n (%) for categorical variables. P <0.05 is statistically significant. BMI data are available in 393 patients.

RAS, renin-angiotensin system; ESA, erythropoiesis-stimulating agents.

Supplementary Table 2. Reasons for cessation of extended-hours hemodialysis

| **Reasons** | **n (%)** |
| --- | --- |
|  |  |
| Wish to switch to conventional dialysis (personal preference) | 39 (36.1%) |
| Kidney transplantation or home dialysis | 16 (14.8%) |
| External factors (disaster, family issues, or relocation to other regions) | 18 (16.7%) |
| Internal factors (the requirement for nursing care, difficulty in maintaining regular visits, or transfer to another facility owing to acute illness) | 35 (32.4%) |
| Total | 108 |


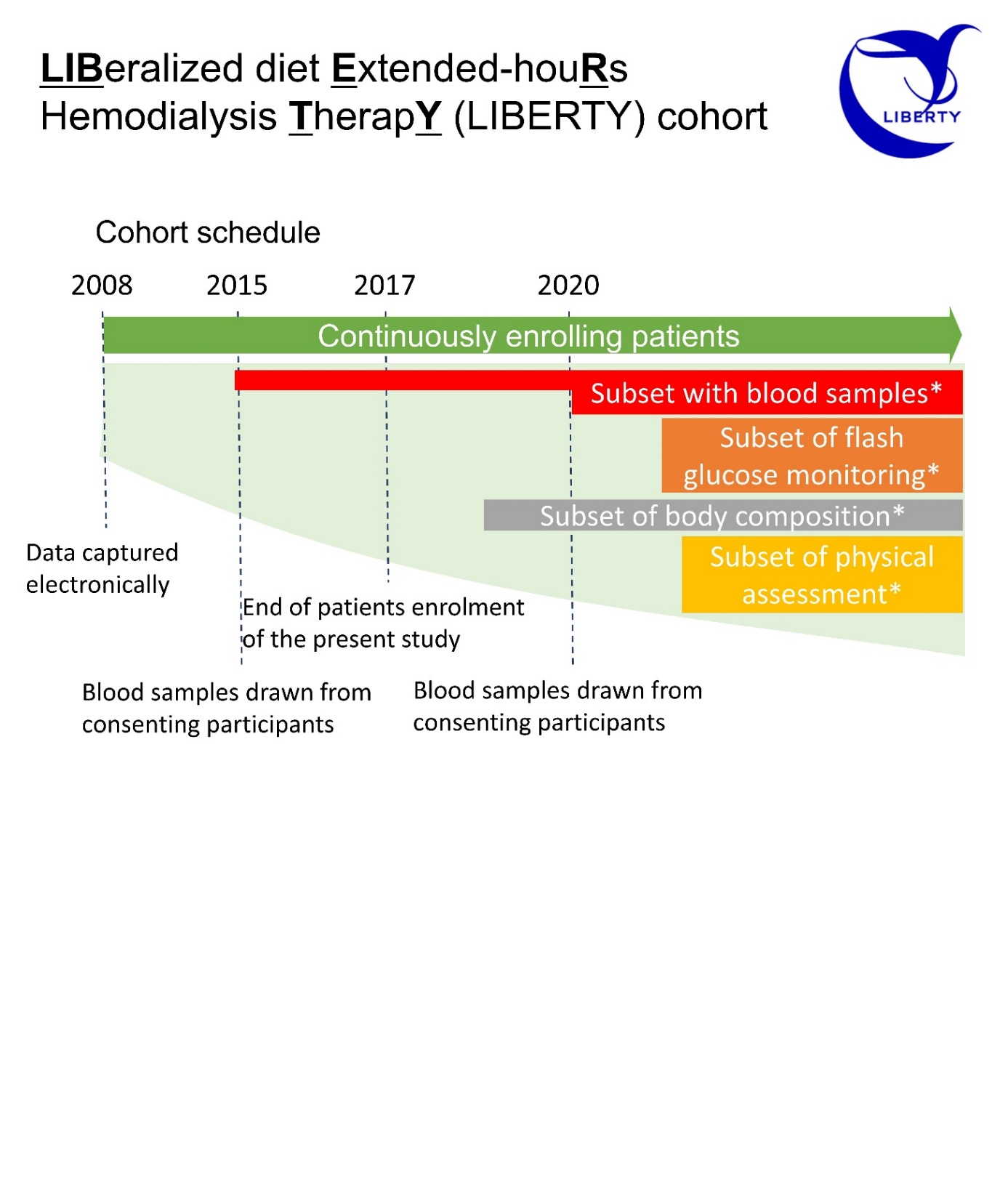
Supplementary Fig 1. Schedule of the LIBERTY cohort

Clinical data including dialysis conditions, prescription, and routine laboratory results were captured electronically and then anonymized. The demographic and outcome data were collected and managed using Research Electronic Data Capture (REDCap) electronic data capture tools hosted at Nagoya University Hospital. REDCap is a secure web-based software platform that supports data extraction for research studies. It provides an intuitive interface for validated data capture, audit trails for tracking data manipulation and export procedures, automated export procedures for seamless data download to common statistical packages, and procedures for data integration and interoperability with external sources [S1,2]. (*Participants in the subsets of flash glucose monitoring, body composition, blood samples, and physical assessment provided written informed consent. Written informed consent was waived in studies only used electronically captured anonymized data.)

[Reference]

S1. Harris PA, Taylor R, Thielke R, Payne J, Gonzalez N, Conde JG. Research electronic data capture (REDCap)-A metadata-driven methodology and workflow process for providing translational research informatics support. J Biomed Inform. 2009;42:377–81

S2. Harris PA, Taylor R, Minor BL, Elliott V, Fernandez M, O'Neal L, et al. The REDCap consortium: Building an international community of software platform partners. J Biomed Inform. 2019;95:103208.

Supplementary Fig 2. Flow diagram


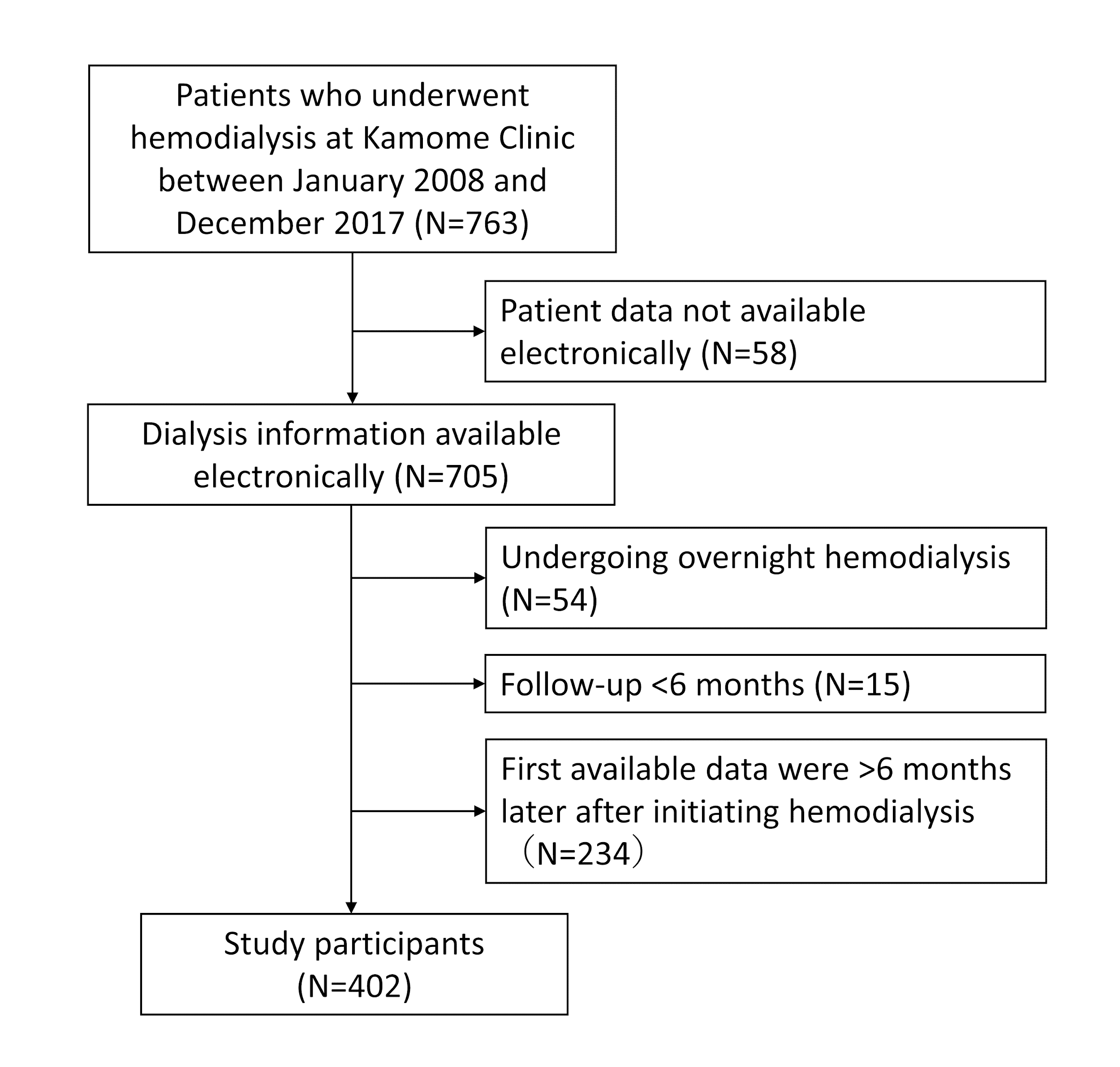


Supplementary Fig 3. Longitudinal changes in body mass index stratified by duration of conventional dialysis


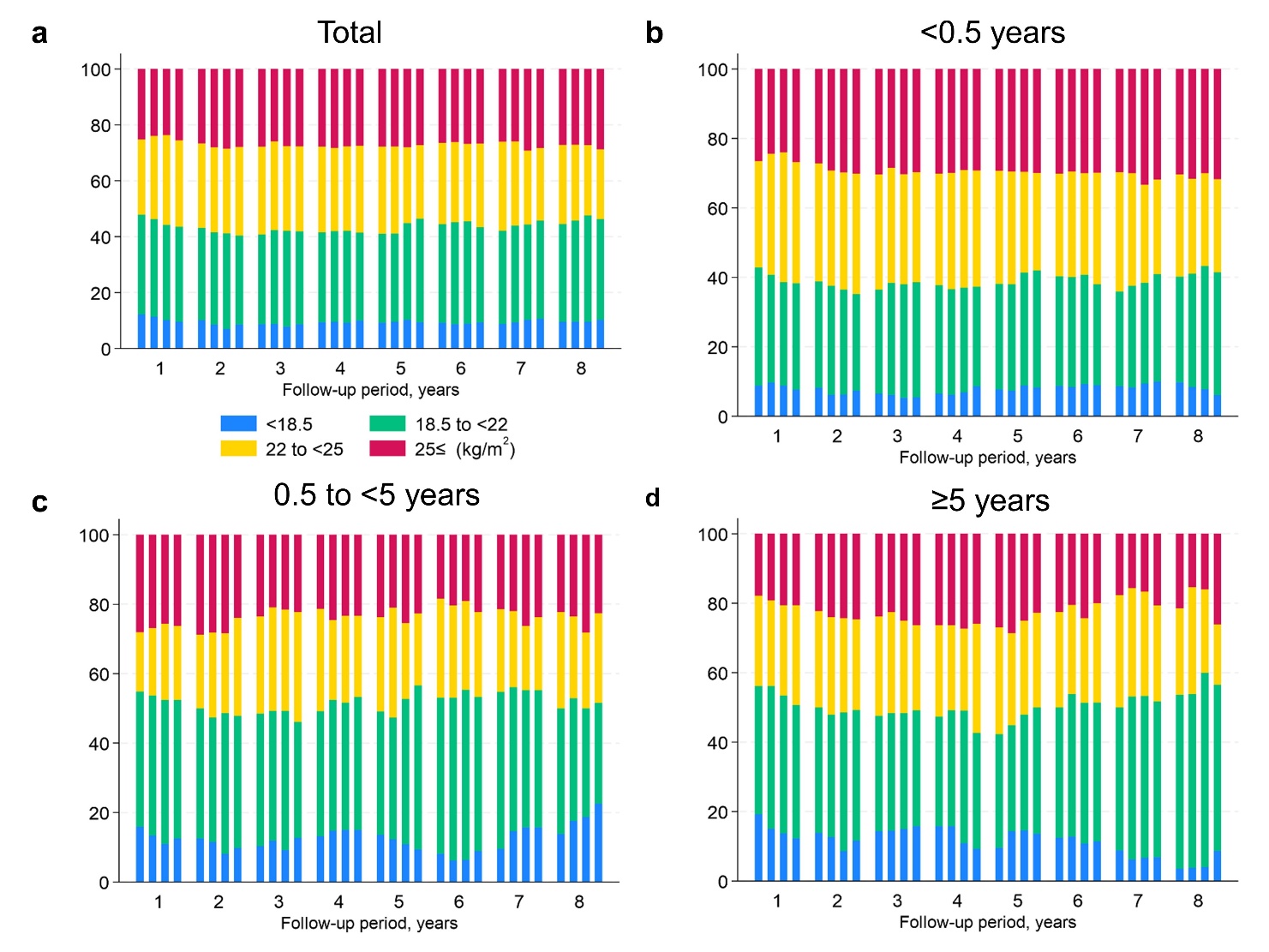


**a.** Total (N=393). Patients who had been on conventional dialysis for <0.5 years, 0.5 to <5 years, and ≥ 5 years (**b**, N=238; **c**, N=82; d, N=73). Body mass index remained stable over time even in patients who had been on conventional dialysis for ≥5 years.

Supplementary Fig 4. Longitudinal changes in the number of antihypertensive agents


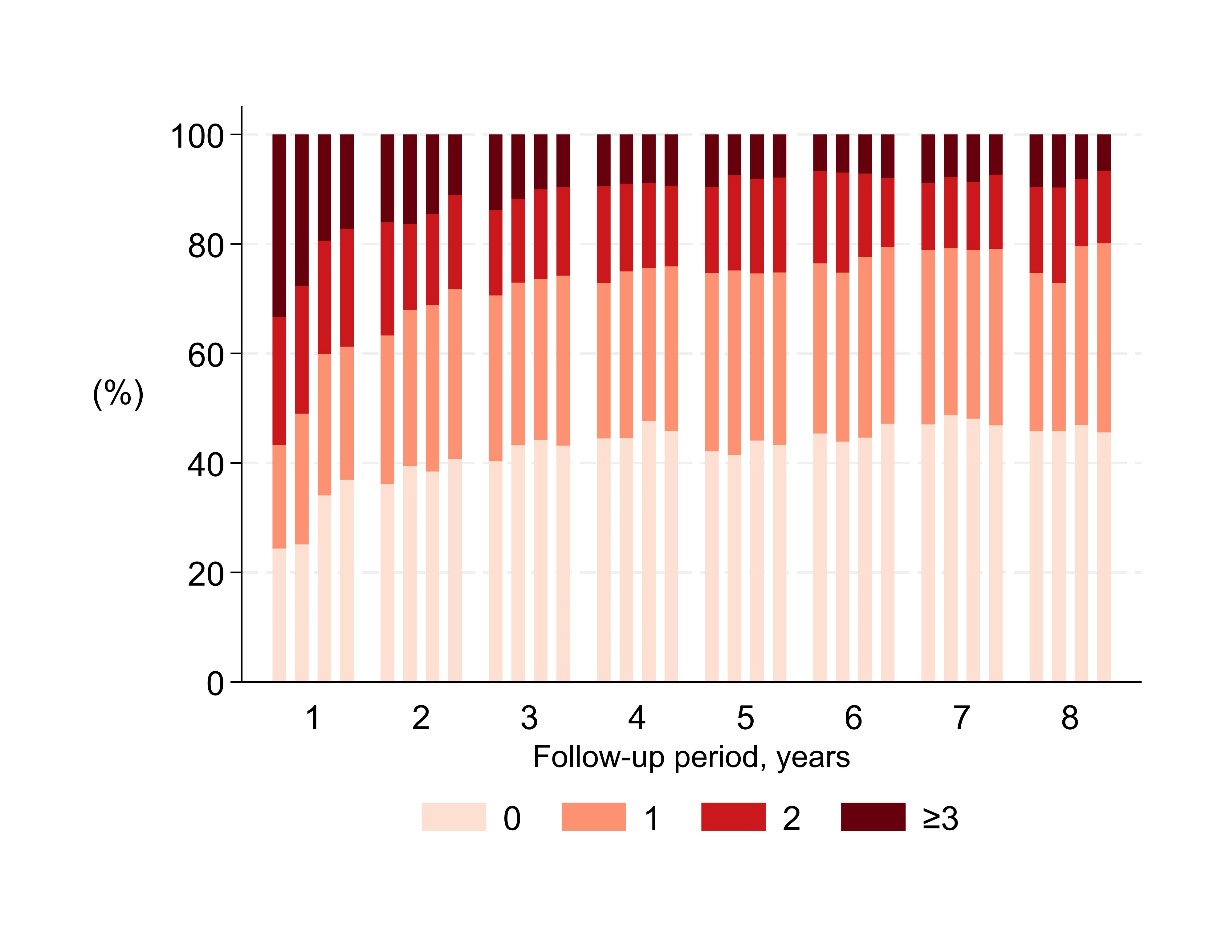


Each bar represents a quarterly category for each year through 8 years. Quarterly medications were defined as those filled at least once in each quarter. The number of antihypertensive agents decreased over time.

Supplementary Fig 5. Longitudinal change in erythropoiesis-stimulating agent resistance index

Each violin plot represents the distribution of quarter means for each year up to 8 years. ERI does not largely unchanged over time. ESA, erythropoiesis-stimulating agent; ERI, ESA resistance index.

Supplementary Fig 6. The estimated trajectories of body mass index across different strata


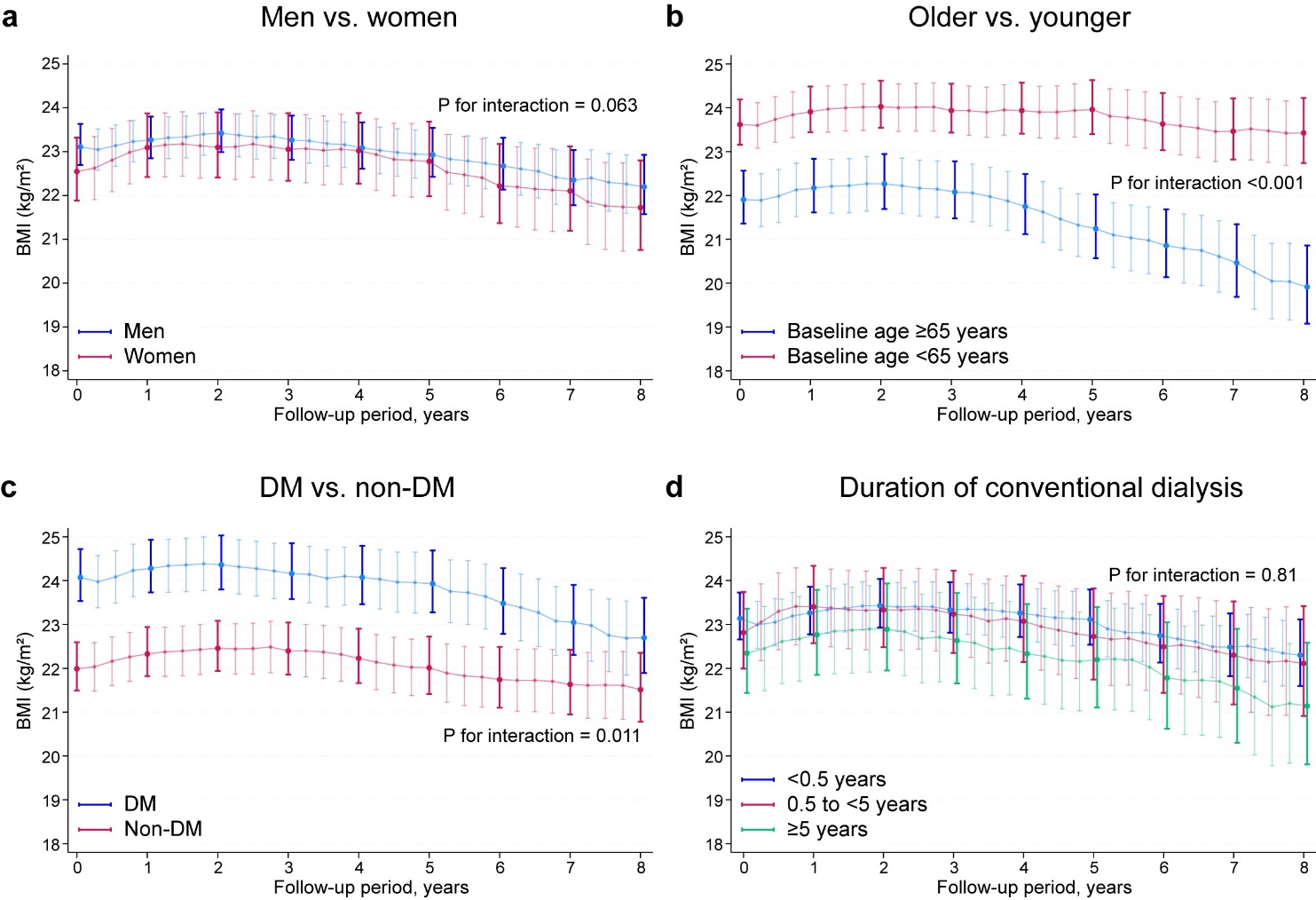


The estimated trajectories of BMI stratified by men vs. women (**a**, P for interaction= 0.063), older vs. younger (**b**, P for interaction <0.001), DM vs. non-DM (**c**, P for interaction = 0.011), and the duration of conventional dialysis <0.5, 0.5 to <5, ≥5 years (**d**, P for interaction = 0.81). The BMI trajectories were estimated using a mixed-effects model adjusted for covariates: age, sex, DM, and the duration of conventional dialysis, excluding a variable related to the stratum. The BMI levels remained largely unchanged regardless of the duration of conventional dialysis. Younger patients are more likely to retain BMI than older patients. BMI, body mass index; DM, diabetes mellitus.

Supplementary Fig 7. The estimated trajectories of body mass index between deceased and living patients

The BMI trajectories estimated using a mixed-effects model adjusted for covariates: age, sex, DM, and the duration of conventional dialysis were compared between deceased and living patients. The BMI levels remained largely unchanged in living patients, while deceased patients showed a decreasing trajectory of BMI after 2 to 3 years from initiation of extended-hours hemodialysis. BMI, body mass index; DM, diabetes mellitus.

Supplementary Fig 8. The estimated trajectories of percentage creatinine generation rate across different strata


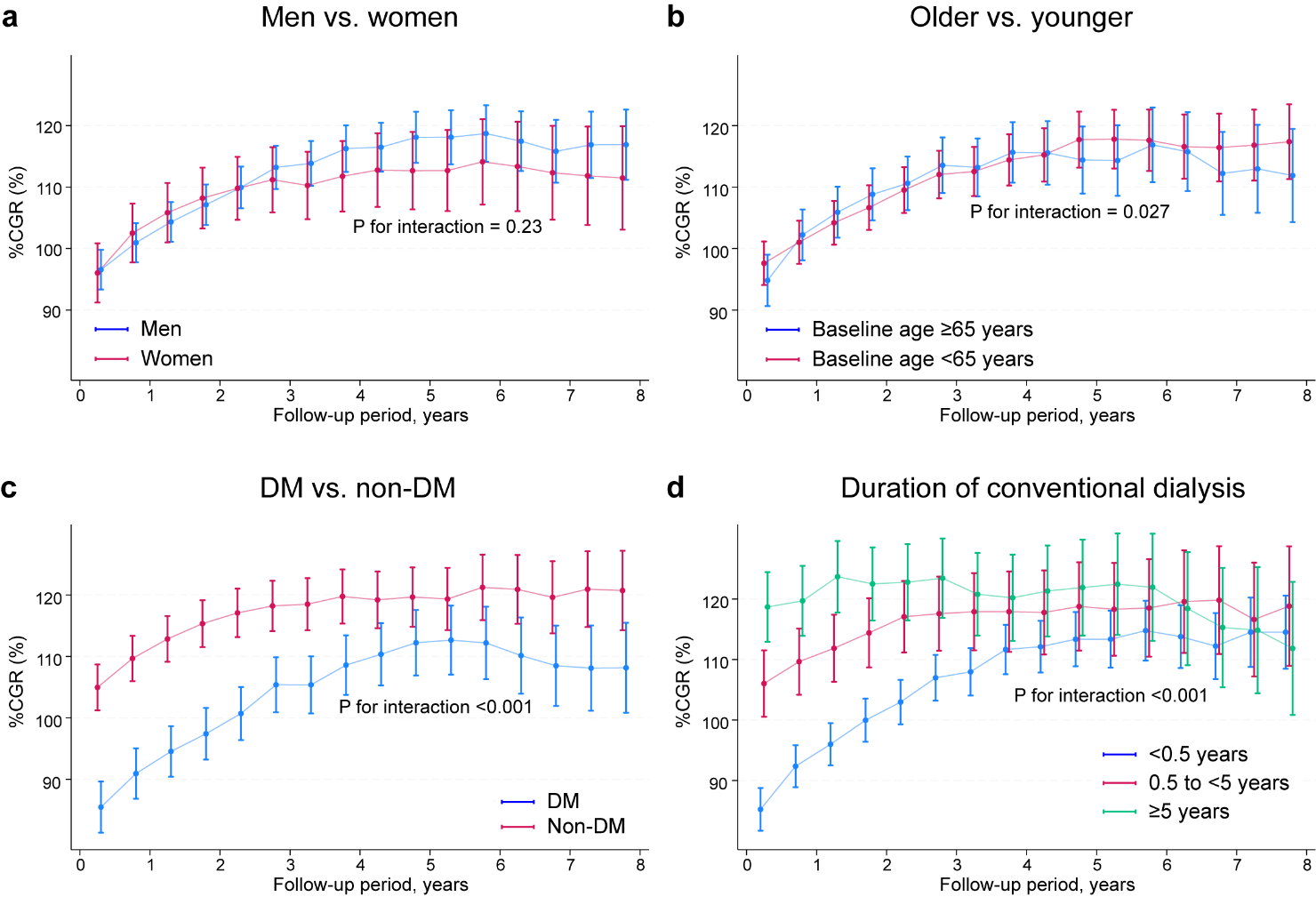


The estimated trajectories of %CGR stratified by men vs. women (**a**, P for interaction= 0.23), older vs. younger (**b**, P for interaction 0.027), DM vs. non-DM (**c**, P for interaction <0.001), and the duration of conventional dialysis <0.5, 0.5 to <5, ≥5 years (**d**, P for interaction < 0.001). The %CGR trajectories were estimated using a mixed-effects model adjusted for covariates: age, sex, DM, and the duration of conventional dialysis, excluding a variable related to the stratum. The %CGR levels increase in the early years after starting extended-hours dialysis especially in patients with shorter length of conventional dialysis. The %CGR levels remained largely unchanged in not only younger patients but also older patients. %CGR, percentage creatinine generation rate; DM, diabetes mellitus.
